# Technical Considerations and Preliminary Experience of Intraprocedural Aneurysm Sac Embolization During Fenestrated and Branched EVAR (Embo F/BEVAR Technique): A Case Series

**DOI:** 10.3390/jcm14082709

**Published:** 2025-04-15

**Authors:** Andrea Xodo, Fabio Pilon, Alessandro Gregio, Giulia Ongaro, Alessandro Desole, Federico Barbui, Giovanni Romagnoni, Domenico Milite

**Affiliations:** Division of Vascular and Endovascular Surgery, “San Bortolo” Hospital-AULSS8 Berica, 36100 Vicenza, Italy; fabio.pilon@aulss8.veneto.it (F.P.); alessandro.gregio@aulss8.veneto.it (A.G.); giulia.ongaro@aulss8.veneto.it (G.O.); alessandro.desole@aulss8.veneto.it (A.D.); federico.barbui@aulss8.veneto.it (F.B.); giovanni.romagnoni@aulss8.veneto.it (G.R.); domenico.milite@aulss8.veneto.it (D.M.)

**Keywords:** coils, aneurysm sac embolization, type 2 endoleak, FEVAR, BEVAR, embo F/BEVAR

## Abstract

**Background:** The aim of this case series is to describe technical considerations and preliminary outcomes of preventive aneurysm sac embolization during fenestrated or branched EVAR (embo F/BEVAR technique). **Methods:** Five male patients suffering from juxtarenal or pararenal abdominal aortic aneurysms, preoperatively identified as being at “high risk” of type 2 endoleak (EL2) development, were treated with embo F/BEVAR. The patients presented at least two of these risk factors: patent inferior mesenteric artery (IMA) > 3 mm; more than three pairs of patent lumbar arteries (LAAs); more than two pairs of LAAs, associated with an accessory efferent artery or at least a pair of intercostal arteries; aneurysm thrombus volume < 40%; aneurysm sac diameter > 65 mm. Embo F/BEVAR was performed with 15 × 20 mm MReye Inconel coils (Cook Medical, Limerick, Ireland), using different aortic endografts. **Results:** Technical success was 100%, with no complications related to perioperative or postoperative coils implantation. An average number of 11 ± 4.4 coils/patient was deployed. No reinterventions were observed during the follow-up (12.4 ± 3.6 months). One case of EL2 (20%) was detected during the follow-up, without aneurysm sac enlargement. **Conclusions:** According to this preliminary experience, embo F/BEVAR technique with Inconel coils seems a feasible adjunctive procedure to manage the risk of EL2 after FEVAR or BEVAR, allowing a simple follow-up with low levels of scatter artifacts, and ensuring limited additional procedural costs. Moreover, embo F/BEVAR can be used with different endografts, requiring minimal increases in operating times. Further studies with larger cohorts of patients and longer follow-up periods are mandatory to better define the potential of this technique and its limitations.

## 1. Introduction

In the last 30 years, endovascular aneurysm repair (EVAR) has reduced the invasiveness of treatment for abdominal aortic aneurysm (AAA) in patients with suitable anatomy [1]. The advantage of this treatment, compared with open surgical repair (OSR), mainly consists in lower perioperative mortality which is associated with shorter periods of hospitalization after surgery [2,3]. However, randomized trials have shown that after a few years this advantage is no longer seen, because of excess late mortality among patients who have undergone EVAR [4].

Endovascular repair presents some peculiar complications. The most frequent indication for reintervention after EVAR is represented by endoleak (EL) [5,6]. In particular, type two EL (EL2) occurs after EVAR in 8–50% of patients.

Most types of EL2 are innocuous, but those with a persistent inflow–outflow mechanism can cause a significant sac enlargement, resulting in clinical “failure” [7,8,9,10].

Regarding complex endovascular aortic repair, including thoracic and thoracoabdominal procedures, different techniques have been proposed to preserve aortic branch patency (supra-aortic trunks or splanchnic vessels), including parallel grafts, fenestrated or branched endografts, in situ fenestrations or “semi-custom” endografts [11,12,13].

Fenestrated and branched EVAR (F/BEVAR) nowadays represents a feasible and widely accepted technical solution to address juxtarenal (JAAAs), pararenal (PAAAs), and thoracoabdominal aortic aneurysms (TAAAs), in which the proximal sealing zone in moved above the renal/visceral vessels, with the need to preserve their patency [14]. Nevertheless, complex F/BEVAR for extensive aortic disease remains a demanding procedure, and intra- and postoperative complications may arise in up to 10–30% of cases [15].

Several reports in the literature describe intraoperative aneurysm sac embolization during EVAR in order to reduce the risk of EL2 [9,16]; however, preventive treatment of this complication during F/BEVAR is poorly described, despite the fact that EL rates after these procedures are not insignificant; in addition, their nature is often unclear, and indications for treatment remain a matter of debate [17]. Furthermore, even in the presence of extensive aortic coverage, most EL2s during FEVAR or BEVAR appear to arise from the infrarenal abdominal aorta [18].

The aim of this paper is to describe our technique and to report our preliminary experience with intraprocedural aneurysm sac embolization during F/BEVAR (embo F/BEVAR) in five patients considered at “high risk” of EL2 development.

## 2. Materials and Methods

### 2.1. Study Design and Patient Selection

A retrospective and physician-initiated study, based on data collected from a single-center institution (Vascular and Endovascular Surgery Department—“San Bortolo” Hospital, AULSS8 Berica, Vicenza, Italy) was designed, in order to report as a “case series” our experience with patients treated with embo F/BEVAR for JAAA or PAAA. 

Only patients who underwent elective procedures were considered for this study. Patients who underwent FEVAR or BEVAR to treat secondary EL1A, patients treated in an emergent or urgent setting, and patients who had previously undergone open abdominal aortic surgery were excluded.

**Figure 1:**
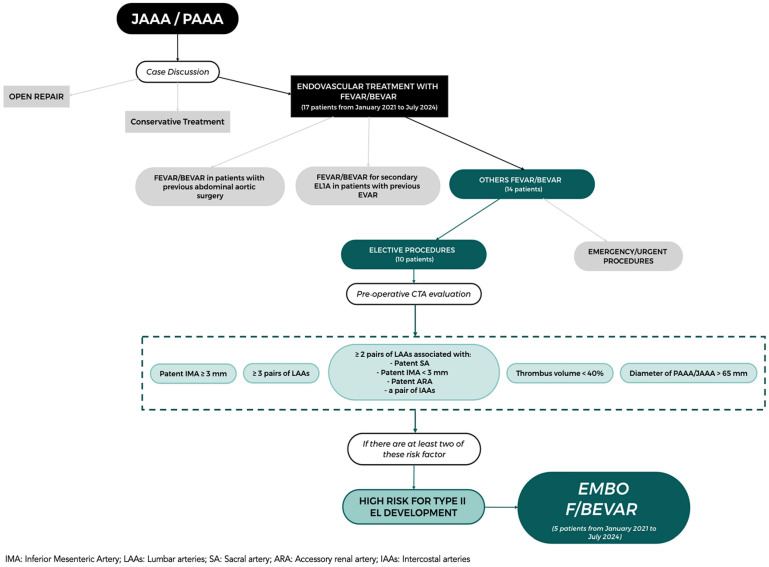
Flow diagram of patient selection before embo F/BEVAR.

From January 2021 to July 2024, 17 patients were treated with F/BEVAR for different clinical conditions (JAAA or PAAA). In 5 (29.4%) patients, the adjunctive intraoperative procedure of aneurysm sac embolization (embo F/BEVAR) was considered (Figure 1).

The indications for embo F/BEVAR were based on anatomic criteria extracted from the available literature [18,19,20]. Patients were therefore considered at high risk for EL2 development and eligible for embo F/BEVAR if they presented at least two of these risk factors: a patent inferior mesenteric artery (IMA) larger than 3 mm; more than three pairs of patent lumbar arteries (LAAs); more than two pairs of LAAs, associated with a patent sacral artery (SA) or IMA/accessory renal artery (ARA) or at least a pair of intercostal arteries (IAAs); aneurysm thrombus volume < 40%; large aneurysm sac diameter (>65 mm).

All these vessels should have been covered by the endografts according to the preoperative planning, to be considered the source of a possible EL2.

All data were obtained from medical records and stored in a dedicated database. The embo F/FEVAR technique consists of a “standard” FEVAR or BEVAR procedure, with the adjunctive intraoperative step of the aneurysm sac embolization using exclusively MReye coils (Cook Medical, Limerick, Ireland); these coils are made of Inconel, an MR Conditional superalloy with spaced synthetic fibers, and are supplied preloaded in a loading cartridge. The coil size was 15 × 20 mm for all patients. This size is the longest available, and guarantees an approximate number of loops of 2.4.

Sac embolization technique during embo F/BEVAR is similar to the procedure used for many years at our institution during EVARs, except for the choice of coils, which was changed in 2018, and an absence of intrasac fibrin glue injection [21].

The endpoints of the study were to assess primary technical success, EL incidence (in particular EL2), reintervention rates during the follow-up (FU), and coil-related complication rates.

Written informed consent for data collection was obtained from all patients, while Institutional Review Board requirements were waived for this retrospective analysis of anonymized data.

### 2.2. Definitions

Pre-operative computed tomography angiograms (CTAs) were analyzed by the same vascular surgeon (A.X.) with dedicated software for center lumen line analysis (3Mensio, e-bi—Version 10.2—Bilthoeven, The Netherlands). Extension of the aortic aneurysms was classified according to reporting standards for endovascular aortic aneurysm repair, based on preoperative CTA [22]. Anatomical characteristics identified at preoperative CTA were aneurysm sac maximum diameter, patency and diameter of the IMA, patency of the SA, and numbers of patent pairs of LAAs and IAAs (Table 1).

Moreover, total aneurysm volume (TAV) and thrombus volume (TV) were calculated using automated and manual features of the software, to obtain a thrombus percentage and to calculate the aneurysm residual luminal volume (RLV) of the aneurysm (Table 2).

To obtain these measurements, we considered the length from the lowest renal artery to the aortic bifurcation for JAAA and PAAA. During the planning step we calculated the true RLV (TRLV) by subtracting the volume occupied by the endograft (approximately the volume of a cylinder with the diameter of the endograft and the length of the aneurysm sac) from the RLV.

Aneurysm sac embolization was performed on all patients with Inconel coils (MReye coils, Cook Medical, Limerick, Ireland). Coils had a straight length of 15 mm and an “embolus diameter” of 20 mm. We calculated the minimum number of coils to obtain an effective embolization using the formula reported by Mascoli et al.: N° eff. Coils = residual volume (cm^3^) × 0.17 [16]. The surgical team could however use a higher or a lower number of coils, compared with the theoretical minimum number of coils, if deemed appropriate to obtain adequate embolization.

Technical success and possible medical and/or surgical complications, including spinal cord ischemia (SCI), were described according to current reporting standards [12].

Postoperative medical therapy consisted of dual antiplatelet therapy (APT) for 30 days (aspirin 75–100 mg daily and clopidogrel 75 mg daily), followed by long-term single antiplatelet therapy with aspirin. If the patient needed anticoagulation, this medication was usually continued, after calculating the risk/benefit ratio for an additional single/dual APT.

Patients were subjected to standard follow-up protocol consisting of a CTA performed within 30–90 days and yearly thereafter. All follow-up CTAs included a portal and a late contrast phase. Follow-up images were analyzed by the same vascular surgeon and compared with the pre-operative CTA using the same software.

Variations in dimensions of the aneurysm sac, as well as EL characterization, were described according to the SVS reporting standards [12].

### 2.3. Technical Procedure for “Standard” FEVAR and BEVAR

General anesthesia was performed for all cases, and all the procedures were performed in a conventional operating room (OR) under fluoroscopy guidance, using a mobile Ziehm C-arm (Ziehm Imaging GmbH, Nurnberg, Germany).

Endoluminal access to the aorta was in all cases performed through a puncture of both common femoral arteries (CFAs) by duplex ultrasound (DUS) or surgical cut-down. An additional surgical access with a direct vessel puncture was performed at the left axillary or brachial artery, in order to allow cannulation of directional branches or fenestrations. For selected cases, the procedure was completely performed only from the femoral accesses, using a steerable sheath to stent the visceral vessels in the case of BEVAR, or a large Dryseal sheath (W. L. Gore & Associates), in standard fashion, in the case of FEVAR.

In cases of JAAA, Fenestrated Anaconda or Fenestrated Treo (Terumo Aortic, Inchinnan, UK) was used as the main endograft; after endograft deployment (Anaconda can be recollapsed and repositioned at the desired location) at least two visceral vessels, usually a renal artery and the superior mesenteric artery (SMA) were cannulated before definitive endograft deployment, in order to ensure a correct alignment.

To gain access to the visceral vessels from below, contralateral limb gate cannulation was performed (by the magnetically linking guidewire in the case of Anaconda, in a standard fashion in the case of Treo); subsequently, a large 18–20 Fr Gore Dryseal (W. L. Gore & Associates, Inc) was advanced inside the endograft and two 7 Fr Flexor sheaths were inserted in a parallel way for vessel cannulation and stenting.

An off-the-shelf Cook Zenith T-Branch (Cook Medical Inc., Brisbane, Australia) multibranch endograft was used to treat a PAAA with a large suprarenal aorta. Usually, a 12 Fr sheath was introduced from above into the descending aorta, followed by an 8 Fr sheath. The main body was advanced from the femoral access and deployed 15–20 mm proximally to the corresponding target vessel, as shown by a precise angiography. A bifurcated graft (Zenith Unibody) with iliac extensions was used if necessary. Bridging stents were usually deployed with the aim of achieving a standard sealing length of 15 mm in the artery [23].

In cases of FEVAR, a protrusion of the balloon-expandable bridging stent-graft into the main aortic graft of 3/5 mm was obtained; after deployment of bridging stents, the proximal edge was systematically flared using a 12 × 20 mm or 10 × 20 mm compliant balloon. For BEVAR procedures, the directional branches were usually stented using a covered self-expandable stent-graft as main bridging stent (only two branches were stented with a covered balloon-expandable stent-graft).

### 2.4. Technical Procedure for Embo F/BEVAR

**Figure 2:**
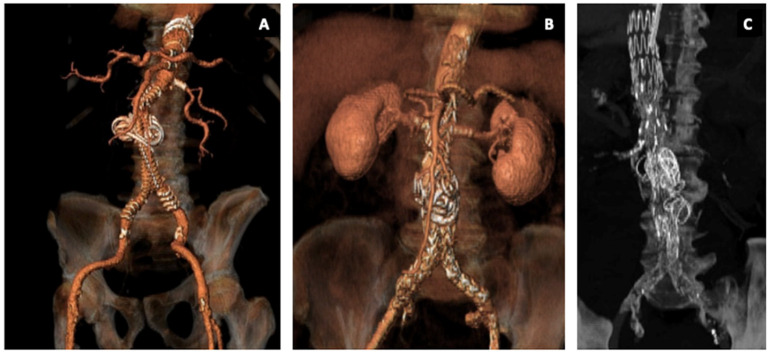
(**A**) 3D reconstruction after embo FEVAR with Anaconda Fenestrated Endograft. (**B**) 3D reconstruction after embo FEVAR with Treo Fenestrated Endograft. (**C**) 3D reconstruction after embo BEVAR with Zenith Cook t-branch device.

When Fenestrated Anaconda (Figure 2A) or Fenestrated Treo (Figure 2B) was used as the main endograft (Embo FEVAR), in order to embolize the sac, a 4 Fr Berenstein catheter (Cordis, Miami, FL, USA) was inserted over a standard 0.035″ hydrophilic guidewire into the aneurysm sac, prior to the iliac limb deployment (usually the contralateral).

In cases of Embo BEVAR with Cook Zenith T-Branch (Figure 2C), the embolization procedure was performed after the bifurcated Unibody component deployment; prior to contralateral limb insertion, a 4 Fr Berenstein catheter was advanced into the aneurysm sac in a similar fashion to the Embo FEVAR procedure.

If a distal extension with a bifurcated component was not required, sac embolization was performed through a 4 Fr Bernstein catheter advanced from the contralateral femoral access into the sac over a standard J-tip 0.035-inch hydrophilic guidewire, before the main graft was deployed.

With both T-Branch or Terumo endografts, care was taken in positioning the tip of the Berenstein catheter near to the origin of the IMA or LAAs, considered the source of EL2. After deployment of ipsilateral and contralateral limbs, contrast was injected into the aneurysm sac to perform a “saccogram” and to confirm the position of the catheter.

Sac embolization was performed with 15 cm × 20 mm coils, and their progression along the tip, under fluoroscopy guidance, was accomplished by a 0.035-inch Emerald Guidewire (Cordis, Miami, FL, USA), in order to facilitate pushability.

After an adequate number of coils was delivered, the Berenstein catheter was removed, and the proximal and distal sealing zones were dilatated with a compliant molding balloon (Reliant, Medtronic, Galway, Ireland). It is crucial to obtain an antero-posterior and a latero-lateral fluoroscopy before placement of coils, in order to confirm the adequate positioning of the catheter and to avoid accidental placing of coils into the visceral vessels.

## 3. Results

### Embo F/BEVAR Case Descriptions and Preliminary Results

**Case 1:** an 80-year-old male presented with an asymptomatic 70 mm juxtarenal abdominal aortic aneurysm. Relevant history included a previous right surgical nephrectomy. A custom-made fenestrated Anaconda bifurcated graft was planned, with three fenestrations for CT, SMA and left renal artery. Pre-operative CTA analysis showed the patency of IMA (3.5 mm), SA, and six pairs among LAAs and IAAs originating from the aneurysm sac. Percentage of thrombus was low (12%). The theoretical minimum effective number of coils was calculated at 36. The procedure was conducted without complications and embolization was performed as described above using 20 MReye coils (15 × 20 mm) for a good filling of the aneurysm sac. Post-operative controls by contrast-CT at 30 days and at 8 months showed an EL2 with an “endoleak circuit” among IMAs, sacral arteries and lumbar arteries, however, without a sac enlargement.

**Case 2:** a 77-year-old male was affected by a 57 mm infrarenal abdominal aortic aneurysm associated with a 40 mm penetrating aortic ulcer located at the origin of the renal arteries. For treatment, a custom-made fenestrated Anaconda bifurcated graft was planned, with four fenestrations. Pre-operative CT analysis showed an aneurysmatic sac with moderate thrombus (45%), a patent IMA (3.5 mm) and 12 afferent vessels (three pairs of LAAs). We planned an intraoperative embolization with a minimum of seven coils. Embolization was performed from the ipsilateral side through a 4 Fr Berenstein catheter located near the IMA origin. A total of 10 MReye coils (15 × 20 mm) were placed in the aneurysmatic sac. No endoleaks were detected at the contrast CT performed 1 month after surgery; after one year, a significant sac shrinkage was detected on a new CTA.

**Case 3:** a 62-year-old male affected by a 66 mm asymptomatic pararenal abdominal aortic aneurysm, without relevant past medical history. An off-the-shelf Cook Zenith T-Branch endograft (four branches) associated with distal deployment of bifurcated Unibody and iliac limbs was planned. Pre-operative CT analysis showed patency of three pairs of LAAs, four pairs of IAAs, and the IMA (<3 mm), resulting in 15 afferent vessels with a moderate–high percentage of thrombus in the aneurysmatic sac (67%). Embolization was performed using a 4 Fr Berenstein catheter positioned in the aneurismatic sac through the ipsilateral side before limb deployment, releasing the minimum number of effective coils for this case (16 MReye coils—15 × 20 mm). The procedure was carried out without complications. Post-operative CT analysis at 30 days and one year later showed an initial sac shrinkage, with no endoleaks.

**Case 4:** a 68-year-old male suffering from a 59 mm asymptomatic juxtarenal abdominal aortic aneurysm who had a positive history for coronary artery disease. The patient underwent endovascular repair using a custom-made fenestrated Anaconda bifurcated graft with four fenestrations. Pre-operative CT scan showed the presence of a large IMA (4 mm), along with a moderate–high percentage of thrombus (70%), patency of two pairs of LAAs and two pairs of IAAs, and a patent SA. The minimum number of effective coils for these characteristics was five. The procedure was carried out without complications. A total of six MReye coils (15 × 20 mm) were released into the aneurismatic sac with a 4 Fr Berenstein catheter trough the ipsilateral access. The final angiogram showed the correct positioning of the main graft and bridging stents without endoleaks. The angio-CT after 18 months confirmed the absence of endoleaks and a stable sac diameter.

**Case 5:** an 80-year-old diabetic male affected by a 61 mm asymptomatic infrarenal abdominal aortic aneurysm associated with a 35 mm penetrating aortic ulcer located at the renal arteries’ origin. Pre-operative CT scan showed the presence of a suitable proximal landing zone between the SMA and the proximal renal artery. Thus, a custom-made bifurcated graft with two fenestrations for renal arteries was planned (Treo, Terumo Aortic, Somerset, NJ, USA). Analysis of the aneurysmatic sac revealed a low percentage of thrombus (38%), a patent IMA and three pairs of afferent LAAs, along with a pair of IAAs. We planned an intraoperative embolization with a minimum of eight MReye coils (15 × 20 mm) which were deployed in the aneurysm sac. Embolization was performed from the contralateral side through a 4 Fr Berenstein catheter. An angiogram at completion and a CTA performed one year after the procedure showed no endoleaks.

In summary, primary technical success was 100% for all patients who underwent embo F/BEVAR. The ELs rate, after a medium CTA FU of 12.4 ± 3.6 months, was 20% (one patient), with no sac enlargement. No coil-related reinterventions or EL2 reinterventions were necessary during the study period.

## 4. Discussion

Endoleaks are a well-known complication after EVAR, and incomplete exclusion of the aneurysm sac can result in persistent sac pressurization, causing continued aneurysm growth and increasing the risk of rupture.

Among different types of endoleaks, EL2 is the result of a retrograde flow into the aneurysm sac from collateral vessels. This “enigmatic” complication has been extensively described in the literature, with not always univocal evolution and clinical implications [24].

EL2 is not “per se” a clinical problem, and is usually generally innocent; however, reintervention rates due to this unwanted event are approximately 10% after EVAR. For this reason, some authors prefer to prevent its incidence [25,26].

A variety of techniques to re-establish the complete aneurysm sac exclusion have been well described, such as transarterial and translumbar approaches, involving IMA/LAAs or a direct puncture of the sac, respectively [27]. Other embolic devices commonly used to embolize the sacs include peripheral vascular embolization coils, cyanoacrylate or fibrin glue, gelfoam, and Onyx [28,29].

However, the incidence and the relevance of EL2 during F/BEVAR has been less widely reported, perhaps as a result of a major focus on target vessel (TV) complications. Oderich and colleagues reported an incidence of isolated EL2 after F/BEVAR of 46% in a group of 184 patients treated for PAAA or TAAA, with a reintervention rate of 10.5% [18].

In our experience with standard FEVAR or BEVAR the rate of this complication was similar (39%); this datum led us to attempt to reduce this percentage by applying the concept of sac embolization, routinely used during EVAR, to FEVAR or BEVAR as well.

In fact, the attention to EL2 should be further emphasized for FEVAR or BEVAR, as this event could lead to a change in sac diameter and theoretically to a “vessel instability”, with the risk of type 3C or 1C for the bridging stents. Squizzato et al. reported that a primary EL2 during FEVAR or BEVAR was associated with failure of aneurysm to regress, after a retrospective analysis on 142 patients treated with fenestrated or branched endovascular aortic repair [17]. Among the available embolization methods, coil embolization has gained widespread acceptance due to its accessibility, effectiveness and relative ease of use [30].

**Figure 3:**
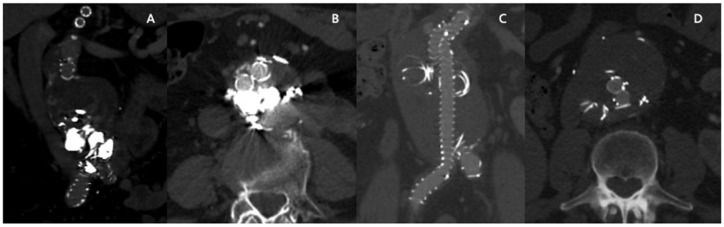
(**A**,**B**) Artifacts in CTA after EVAR with sac embolization by platinum coils. (**C**,**D**) CTA shows clear images and sac high definition after embo FEVAR with MReye deployment.

The use of Inconel coils has some undoubted advantages, such as low cost and easier FU with CTA. Compared to platinum coils, which make follow-up with CTA very difficult due to the presence of numerous artifacts (Figure 3A,B), Inconel coils allow easy visualization of the aneurysm sac and of any new sources of EL (Figure 3C,D).

The price of MReye coils represents a small share of the overall cost of a FEVAR or BEVAR procedure. In our institution, a single Inconel coil costs approximately USD 60 (USD 600–1200 for the deployment of 10–20 coils), compared to controlled detachable platinum coils, which cost around USD 300 (USD 3000–6000 for the same procedure with 10–20 coils).

In our experience, both with embo EVAR or embo F/BEVAR, we have employed long coils (15 cm of extended embolus length and 20 mm of coiled embolus diameter), in order to facilitate the occlusion of large spaces into the sac, close to the high-flow vessels. The downside of MReye is the lack of a system for a controlled release. This may theoretically increase the risk of embolization towards periphery or TVs, compared to controlled release coils [31]; however, this complication was not observed in our case series.

Moreover, adjunctive sac embolization during FEVAR and BEVAR procedures could also potentially increase the risk of SCI, especially in the case of BEVAR for TAAA, due to the deployment of coils at the level of intercostal vessels [32]. This is one of the most severe and devastating complications of complex thoracic and thoracoabdominal aortic procedures; nevertheless, no events of permanent SCI occurred in the group of patients receiving Embo F/BEVAR. SCI risk is only theoretically increased with embo F/BEVAR technique, considering the inevitable aortic coverage in that segment; furthermore, several protocols are available to reduce the risk of spinal injury [33].

**Figure 4:**
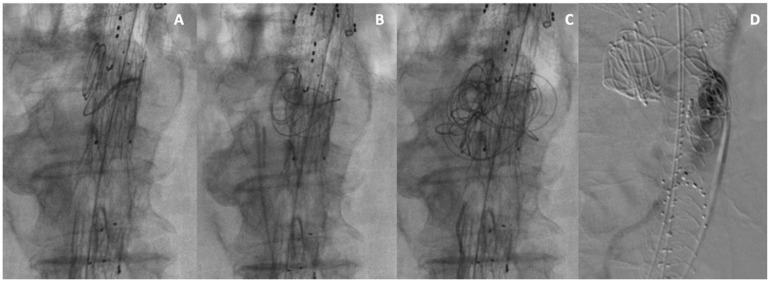
(**A**–**C**) Intraoperative images of sac embolization during embo BEVAR. (**D**) A “saccogram” performed during embo FEVAR with Anaconda endograft.

In our experience, deployment of coils into aortic aneurysm sacs during FEVAR or BEVAR was relatively straightforward after a short learning curve, and the time required (10–15 min) did not extend the procedure or the fluoroscopy time beyond a reasonable length. Immediately after the coils’ deployment (Figure 4A–C), a double radiological projection (anteroposterior and lateral) was used to ensure coils correct positioning, allowing immediate bailout maneuvers in case of coils migration or malpositioning.

The use of a 0.035-inch Emerald Guidewire (Cordis, Miami, FL, USA) to introduce the coils guarantees excellent visibility of the coils and good pushability. The embo F/BEVAR technique does not require selective cannulation of LAAs, IMA or SA, and does not significantly interfere with the F/BEVAR procedure in terms of operative time, dose of iodine contrast, or costs related to adjunctive devices employed, except for the cost of the coils. A “saccogram” (Figure 4D) can be useful to document flow in the LAAs or in the IMA and to show a “non stagnant” flow, which may be an indicator of an endoleak “circuit” [34].

New materials such as ultra-low-density polyurethane shape-memory polymer (SMP) are now available as vascular plugs [35]. Masmann et al. recently described their experience with 18 patients treated with EVAR or TEVAR and intraoperative aneurysm sacs embolization with SMP [36]. All evaluable patients during the FU exhibited a sac regression, despite six patients still presenting an EL2. Similarly to Inconel coils, this device also allows satisfying visibility of a possible EL on CTA, without particular artifacts.

Regarding our preliminary results, in one patient (20% of cases), we found an EL2 despite preventive embolization. This complication was probably due to the high number of afferent vessels from the sac including a large IMA, a low percentage of thrombus, and the large diameter of the aneurysm. In these cases, selective embolization of the IMA associated with a more aggressive sac embolization (a lower number of coils than theoretically expected was used) could probably be considered, to reduce the formation of a persistent inflow–outflow mechanism.

### Study Limitations

There are many limitations in this paper: our results, due to the small number of patients analyzed (case series) and the observational nature of the study, should be interpreted carefully.

This preliminary experience of embo F/BEVAR was gained only recently, and the FU time is short (12.4 ± 3.6 months). In addition, the heterogeneity of treated pathologies (JAAA and PAAA) could also have had a major impact on outcomes, precluding overall conclusions. Nonetheless, this study does address an important issue that has never been scrutinized before, namely, the application of intraoperative aneurysm sac embolization during fenestrated and branched EVAR.

## 5. Conclusions

From this preliminary single-center experience, the use of Inconel coils for intraoperative aneurysm sac embolization during FEVAR or BEVAR (embo F/BEVAR technique) appears to be a feasible and safe method to counteract the risk of EL2, allowing easy visualization of the aneurysmal sac and possible new sources of EL.

In addition, the embo F/BEVAR technique can be used with different types of endografts, requiring minimal increases in procedure costs and operative times. Further studies with larger patient cohorts and longer FUs are needed, to better define the potential and limitations of this technique.

## Figures and Tables

**Table 1:** Summary of risk factors for type 2 EL development and follow-up outcomes in patients who underwent embo F/BEVAR.

	No. of Risk Factors	Description	Total Number of Afferent Vessels	FU Months	Endoleaks Detected/ Sac Expansion
Case 1	5	- 12% thrombus - Patent IMA (3.5 mm) - 4 pairs of LAAs - 2 pairs of IAAs + SA - 70 mm aneurysm sac	14	8	Type 2 EL, no sac expansion
Case 2	3	- 45% of thrombus - Patent IMA (3.5 mm) - 3 pairs of LAAs - 2 pairs of IAAs + 1 ARA - 57 mm aneurysm sac	12	12	No ELs
Case 3	3	- 67% of thrombus - Patent IMA (<3 mm) - 3 pairs of LAAs - 4 pairs of IAAs - 66 mm aneurysm sac	15	12	No ELs, sac shrinkage
Case 4	2	- 70% of thrombus - Patent IMA (4 mm) - 2 pairs of LAAs + SA - 2 pairs of IAAs - 59 mm aneurysm sac	10	18	No ELs
Case 5	2	- 38% thrombus - Patent IMA (3 mm) - 3 pairs of LAAs - 1 pair of IAAs - 61 mm aneurysm sac	9	12	No ELs

IMA—inferior mesenteric artery; LAAs—lumbar arteries; IAAs—intercostal arteries; SA—sacral artery; ARA—accessory renal artery.

**Table 2:** Aneurysms’ characteristics in terms of total aneurysm volume, residual luminal volume, endograft volume and residual true luminal volume; percentages of intraluminal thrombus, with theoretical and real total numbers of coils employed for embo F/BEVAR.

	TAV (cm^3^)	RLV (cm^3^)	Thrombus %	EV (cm^3^)	RLTV (cm^3^)	N° of Coils Planned *	N° of Coils Implanted
Case 1	309	270	12	55	215	36	15
Case 2	128	70	45	30	40	7	10
Case 3	435	142	67	51	91	16	16
Case 4	242	75	70	44	30	5	6
Case 5	162	66	40	51	45	8	8

TAV—total aneurysm volume; RLV—residual luminal volume; EV—endograft volume; RTLV—residual true luminal volume; * (concentration of 0.17 coils/cm^3^).

## Data Availability

The original contributions presented in this study are included in the article. Further inquiries can be directed to the corresponding author.
